# European multicenter study on recurrent imaging practice in computed tomography

**DOI:** 10.1093/bjr/tqag134

**Published:** 2026-06-04

**Authors:** Marco Brambilla, Jenia Vassileva, Simona Avramova-Cholakova, László Balkay, Efstathios P Efstathopoulos, Kathrin Entz, Hannie Förnvik, Felix Gebser, Emil Georgiev, Bouchra Habib Geryes, Birute Griciene, Joanna Kidoń-Szołtysek, Marta Kolarek, Mika Kortesniemi, David Kozák, Ivana Kralik, Ingunn Løvik, Georgios Menikou, Roberto M Sánchez, Marta Sans Merce, Alexander Schegerer, Marcus Söderberg, Annalisa Trianni, Janne Vignero

**Affiliations:** Medical Physics Department, A.O.U. “Maggiore della Carità”, Novara, Italy; Radiation Protection Consultant, Vienna, Austria; Charing Cross Hospital, Imperial College Healthcare NHS Trust, London, United Kingdom; Department of Nuclear Medicine and Translational Imaging, Institute of Medical Imaging, Faculty of Medicine, University of Debrecen, Debrecen, Hungary; Department of Applied Medical Physics, School of Medicine, National and Kapodistrian University of Athens, Greece; Clinic for Radiology and Nuclear Medicine, Hospital of the Goethe University Frankfurt, Frankfurt am Main, Germany; Radiation Physics, Department of Hematology, Oncology and Radiation Physics, Skåne University Hospital and Lund University, Malmö, Sweden; University Medical Center Göttingen, Institute for Clinical and Interventional Radiology, Göttingen, Germany; Radiology Department, Acibadem City Clinic University Hospital Tokuda, Sofia, Bulgaria; Pediatric Radiology Department, Université Paris Cité, AP-HP, Hôpital Universitaire Necker Enfants Malades, Paris, France; Faculty of Medicine, Vilnius University, Vilnius University Hospital Santaros Klinikos, Vilnius, Lithuania; Unit of Invasive Cardiology and Electrocardiology, 3rd Department of Cardiology, Medical University of Silesia, Katowice, Poland; Department of Medical Physics, University Hospital Centre Zagreb, Zagreb, Croatia; HUS Diagnostic Center, Radiology, Helsinki University and Helsinki University Hospital, Helsinki, Finland; Department of Medical Physics and Radiation Protection, University Hospital Olomouc, Olomouc, Czech Republic; Clinical Department of Diagnostic and Interventional Radiology, Dubrava University Hospital, Zagreb, Croatia; Department of Radiology and Nuclear Medicine, St. Olavs hospital, Trondheim University Hospital, Trondheim, Norway; Limassol General Hospital, State Health Services Organization, Limassol, Cyprus; Hospital Clínico San Carlos, IdISSC and Universidad Complutense de Madrid, Madrid, Spain; Diagnostic Department, Geneva University Hospital, Geneva, Switzerland; Hirslanden Mediclinic Group, Corporate Office, , Glattpark, Switzerland; Radiation Physics, Department of Hematology, Oncology and Radiation Physics, Skåne University Hospital and Lund University, Malmö, Sweden; Medical Physics Department, ASUIT, Trento, Italy; Department of Radiology, Leuven University Hospital, Leuven, Belgium

**Keywords:** computed tomography, cumulative effective dose, recurrent imaging, reference level

## Abstract

**Objectives:**

This study compares recurrent CT imaging practices across European centers using incidence and prevalence metrics and evaluates the feasibility of a novel benchmarking tool for recurrent imaging practice.

**Methods:**

CT exposure data were collected from 31 facilities across 18 European countries. The single-year incidence proportion (*I*_100;1_), 3-year cumulative incidence (*I*_100;3_), and 3-year prevalence proportion (*P*_100;3_) of patients with cumulative effective dose (CED) ≥ 100 mSv were calculated and statistically analyzed against relevant indicators. The reference year was 2023, and the 3-year period covered 2022-2024.

**Results:**

The database comprised 711 749 patients in 2023 who underwent 1 658 246 CT exams, with an average of 1.5 ± 0.6 CT exams per patient in this year and 2.4 ± 0.6 exams over 3 years. Across facilities, *I*_100;1_ ranged from 0% to 2.9%, *I*_100;3_ from 0% to 8.6%, and *P*_100;3_ ranged from 0% to 4.4%. A strong linear correlation was found between the third quartile values of yearly CED and *I*_100;1_. Multiple linear relationship was identified between *I*_100;3_, third quartile of yearly CED and percentage of recurrent patients. Third quartiles of yearly CED varied between 7.5 and 29.5 mSv, with the 75th percentile of their distribution 17.6 mSv, lower than in previous studies.

**Conclusion:**

Incidence proportion is preferred for assessing recurrent imaging rates as prevalence substantially underestimates these rates. The 75th percentile of distribution of third quartiles of yearly CED is a reasonable benchmark level to trigger optimization. Investigating the underlying factors and addressing high recurrent rates can contribute to enhanced patient care.

**Advances in knowledge:**

This first European multicenter study showed that the epidemiological index incidence proportion is a better metric to quantify recurrent imaging compared to prevalence. The distribution of early CED values can be used to benchmark recurrent imaging practices and inform patient management strategies.

## Introduction

Global data indicate that CT examinations have increased by 82% over the past decade, now accounting for 61.6% of the collective effective dose.^1^ Interventional radiology and nuclear medicine imaging are also rapidly growing, though their contributions to population dose is comparatively lower. While expanded imaging capabilities benefit patient care, these trends highlight the importance of using these methods responsibly due to the associated radiation risks.

In recent decades, significant efforts have been dedicated to the justification and optimization of individual imaging procedures, with numerous successful outcomes documented in the literature and thoroughly incorporated into regulations and guidelines.^2–6^ Less attention has been paid to the sequencing of imaging exams throughout patient treatment pathways, clinical care bundles and their lifetime. Recent research involving large patient cohorts shows a noteworthy proportion of patients receive cumulative doses over 100 mSv from recurrent CT and other imaging exams.^7–11^ Latest epidemiological studies provide new insight and direct evidence of association of cancer risk and radiation exposure, which supports the focus on doses exceeding 100 mSv, especially if accumulated over a short time period.^12–15^ In response, holistic, patient-centered optimization strategies that address serial imaging have been advocated by international and professional organizations.^16–18^

The first step in every optimization process is to quantify radiation levels delivered to individuals in terms of an appropriate metric, which can be assessed with adequate accuracy and reproducibility. The chosen metric is then used to benchmark practices, indicate the need for review and intervention, and measure outcomes of optimization actions. This forms the foundation of the well-established concept of “reference levels” for optimizing radiological protection, as promoted through international recommendations and safety standards.^2^^,^^5^

Dose metrics used in medical imaging include modality-specific dose indices, eg, CT dose index (CTDI), dose-length-product (DLP), and size-specific dose estimates (SSDE) for CT, which are used in the process of optimization of imaging protocols, to benchmark local practices to Diagnostic Reference Levels (DRLs) and to compare acquisition protocols used in different imaging systems.^4^^,^^19–21^

To characterize the recurrent radiation exposure a patient may experience from multiple imaging studies of different body regions, using different modalities or even different types of radiation, a risk-related metric is required that enables the summation of these different exposures. The current radiological protection system provides 2 such quantities: organ doses and effective dose (ED).^2^^,^^3^^,^^5^^,^^22^ Organ doses are most closely linked to individual detriment and are typically used in epidemiological studies or when assessing personal risk.^2^^,^^5^^,^^12–15^ For most other situations ED, despite its well-known limitations and associated uncertainties, remains the only practical metric that allows for summation as a single value of exposure from different type of examinations performed to a patient.^20^^,^^22–24^ Automatic radiation exposure monitoring system (REMS) available in hospitals offer calculation of ED from the modality specific metrics. This practicality explains why cumulative effective dose (CED) is widely used in studies investigating the frequency and magnitude of recurrent imaging.^7–11^^,^^18^^,^^25^^,^^26^

Most published studies on recurrent imaging report the total number of patients who, over a specified timeframe, reach a particular level of CED—commonly 100 mSv, above which the International Commission on Radiological Protection (ICRP) classifies cancer risk as “moderate,” whereas categories “negligible,” “minimal,” “very low,” or “low” are used for lower dose intervals.^22^ Calculating the proportion of the patients with doses above the chosen CED level relative to all individuals who underwent imaging during the same period yields what is referred to in epidemiology as the “prevalence proportion.”Recent studies by Brambilla et al^25^^,^^26^ suggest that using the incidence proportion—the number of new cases within a specified timeframe—provides a more accurate metric for assessing recurrent imaging than prevalence, which counts both existing and new cases. Their concept relies on a standardized method for calculating ED from modality-specific indexes like DLP for CT, applied at the population level. Additionally, these researchers introduced a new benchmarking index, called “recurrent exposure reference levels,” which they applied at CT facilities in North Italy and Slovakia.^25^^,^^26^

The present study aims to compare recurrent imaging practices across multiple centers in Europe using incidence and prevalence metrics. It also aims to test the feasibility of establishing a new benchmarking tool for recurrent imaging practice across facilities.

## Methods

This was a retrospective observational study, which used summary data from the imaging departments records. Since no identifiable personal data were collected and the planned patient management or treatment was not altered by the study, no patient consent was required.

Medical facilities that performed at least 2000 CT procedures per year and have had an automatic Radiation Exposure Monitoring System (REMS) in place since at least 2022 were eligible to participate. The 3-year period from 2022 to 2024 was selected as the minimum reasonable and statistically robust timeframe for evaluating the methodology, while also facilitating direct comparison with prior research conducted during the same period.^25^^,^^26^ The following information was needed for all CT procedures performed during the period 2022-24: patient demographic data (age, gender), examined anatomical region, total DLP from the examination, and patient unique identifier (ID), which allows for tracking individual exams over the period.

Adult patients who were at least 18 years old at the time of the CT examination in 2023 were included in the analyses at each facility. Patients having a CT in the reference year (2023) and at least one other examination in the same year or in the years 2022 or 2024 were defined as recurrent patients. All diagnostic CTs were included except those of lower limbs, which have a negligible contribution to CT frequency and dose,^1^ as well as CT-guided interventional procedures. CT parts from PET/CT or SPECT/CT were counted, but those for radiotherapy planning or cone-beam CT during treatment were excluded. Multi-phased CT scan acquisitions connected to a single accession number were counted as a single CT exam.

The following standard conversion coefficients from DLP to ED used in the latest global survey^1^^,^^27^^,^^28^ were applied by all facilities: 0.015 mSv/mGy.cm for all exams in the body region (chest, abdomen, pelvis); 0.0021 mSv/mGy.cm for head and head and neck regions, normalized to CTDI in the standard head (16 cm) dosimetry phantom, and 0.0059 mSv/mGy.cm for neck region normalized to CTDI in the standard body (32 cm) dosimetry phantom. Given the variability in ED/DLP conversion coefficients found in the literature and the high associated uncertainties,^27–29^ emphasis in this population-based study was placed on standardizing ED calculation methods across facilities, rather than attempting to refine values. Another reason was the aimed comparison with recently published studies which used the same coefficients.^25^^,^^26^

CED was calculated for each patient who underwent a CT study in 2023 as the sum of ED from all the CT examinations received during a single year (2022, 2023, and 2024) and for the entire 3-year period (2022-24).

Each facility calculated the incidence and prevalence indices defined as follows.

The yearly incidence proportion of patients with CED ≥ 100 mSv in a given year, *I*_100;1_, in percentages_,_ is expressed as:


$$I_{100;\;1}=\frac{N.\;\mathrm{of}\;\mathrm{patients}\;\mathrm{with}\;\mathrm{CED}\geq 100\;\mathrm{mSv}\;\mathrm{in}\;\mathrm{year}\;i}{N.\;\mathrm{of}\;\mathrm{patients}\;\mathrm{receiving}\;a\;\mathrm{CT}\;\mathrm{exam}\;\mathrm{in}\;\mathrm{year}\;i}\;\times 100$$

The 3-year cumulative incidence proportion of patients enrolled in the year 2023 with CED ≥ 100 mSv over 3 consecutive years (2022-2024), *I*_100;3_, in percentages, is expressed as:


$$I_{100;\;3}=\frac{N.\;\mathrm{of}\;\mathrm{patients}\;\mathrm{in}\;2023\;\mathrm{with}\;\mathrm{CED}\geq 100\;\mathrm{mSv}\;\mathrm{in}\;3\;\mathrm{cumulative}\;\mathrm{years}\;(2022-2024)}{N.\;\mathrm{of}\;\mathrm{patients}\;\mathrm{receiving}\;a\;\mathrm{CT}\;\mathrm{exam}\;\mathrm{in}\;2023}\times 100$$

The 3-year prevalence proportion of patients with CED ≥ 100 mSv over 3 consecutive years (2022-24), *P*_100;3_, in percentages, is expressed as:


$$P_{100;\;3}=\frac{N.\;\mathrm{of}\;\mathrm{patients}\;\mathrm{with}\;\mathrm{CED}\geq 100\;\mathrm{mSv}\;\mathrm{in}\;3\;\mathrm{cumulative}\;\mathrm{years}\;(2022-2024)}{N.\;\mathrm{of}\;\mathrm{patients}\;\mathrm{receiving}\;a\;\mathrm{CT}\;\mathrm{exam}\;\mathrm{in}\;3\;\mathrm{cumulative}\;\mathrm{years}\;(2022-2024)}\times 100$$

Data were collected from May to August 2025, analyzed locally by each facility, and reported to study coordinators using a standard Excel template. Descriptive statistics of demographic and exposure data (sex, age, number of CT examinations per patient) were reported for each facility, along with the number of beds in each hospital used to stratify the sample in small (<200), medium (201-500), or large (>500) hospitals.

Following a review by the study coordinators, local data were pooled in a Microsoft Excel database; each facility was assigned a unique number, which was systematically used to present the results. Data were described using mean and standard deviation or median, interquartile ranges and percentiles.

Based on findings of Brambilla et al^25^^,^^26^ that the 75th percentiles (third quartiles) of yearly CED values exhibit a stronger correlation with incident proportions than other percentiles, the third quartiles of yearly CED distributions for the years 2022, 2023, and 2024 were analyzed. This approach was applied to benchmark local practices and examine their correlations with incidence and prevalence indices. The 75th percentile of the pooled third quartiles of yearly CED distribution was calculated to compare with the recurrent exposure reference level (RERL) values from the previous 2 studies.^25^^,^^26^

Correlations between variables were quantified using the Spearman *ρ* coefficient or the Pearson’s *r* coefficient depending on distributional verifications. Temporal trends in the average number of patients, number of CT and *I*_100;1_ over 3 years were assessed using a repeated measures ANOVA.

Box and whiskers plots were used to provide a univariate graphical representation of the epidemiological indexes *I*_100;1_, *I*_100;3_, and *P*_100;3_. Outliers and extremes are points higher than the value of the third quartile plus 1.5 or 3× the interquartile distance, or lower than the value of the first quartile minus 1.5 or 3× the interquartile distance, respectively.

Linear and polynomial regression studies were conducted between the epidemiological variable *I*_100;1_ and the third quartile of yearly CED distributions. The impact of the different statistical CED descriptors over 1 year as well as the percentage of recurrent patients on *I*_100;3_ was assessed by multiple linear regression models with forward stepwise selection of variables (*F* to enter = 4, tolerance = 0.001). Standardized regression coefficients were used as a measure of relative importance of the predictors in explaining the variance of the dependent variable *I*_100;3_. The Kolmogorov-Smirnov test was used to check the normality of residual distributions, which is required for performing parametric tests of significance.

Surface plots with least square fitting were used to provide a bivariate graphical representation of the dependent variables with respect to significant predictors. The models obtained should not be extrapolated outside the ranges of predictor values for which they have been determined.

Statistical analyses were performed using the software STATISTICA 11 (StatSoft Inc) using a 2-sided type I error rate of *P *= .01.

## Results

Thirty-one facilities from 18 European countries participated in the study. Their distribution by country is as follows: Belgium (1), Bulgaria (1), Croatia (2), Cyprus (1), Czech Republic (1), Finland (1), France (1), Germany (2), Greece (1), Hungary (1), Italy (1), Lithuania (1), Norway (3), Poland (1), Spain (1), Sweden (9), Switzerland (2), and the UK (1). By type, there were 17 university, 13 general, and 1 specialized hospitals. Among them, 8 were classified as small, 4 as medium, and 19 as large hospitals. The accuracy of dose display was tested annually in each facility with a maximum tolerance of 20%.

The database included 711 749 patients in the reference year who underwent 1 658 246 CT exams, with an average of 1.5 ± 0.6 CT exams per patient in the reference year and 2.4 ± 0.6 exams per patient over 3 years.

Table 1 reports for each facility the number of patients in 2023, their sex distribution, descriptive statistics of age, number and percentage of recurrent patients, as well as the number of patients with CED ≥ 100 mSv over 3 years. Table 1 also shows *I*_100;1_ in the reference year, and the following data for 3 calendar years: number of exams, *I*_100;3_, *P*_100;3_, descriptive statistics of CED and number of CT exams per patients. The last column in Table 1 shows the yearly CED for the 3 years.

**Table 1:** Descriptive statistics of number of patients, sex distribution, age, and *I*_100;1_ in the reference year (2023) in different facilities, as well as *I*_100;3_ and *P*_100;3_, number of patients with CED ≥ 100 mSv, descriptive statistics of CED in 3 years (denoted as CED_3_) and number of exams in different facilities in 3 years.

Facility #	No. of patients in 2023	% Female	Age (years), median [IQR]	No. exams in 2022, 2023, 2024	No. (%) of recurrent patients	No. of patients with CED_3_ ≥ 100 mSv	*I*_100;1_ (%) in 2023	*I*_100;3_ (%)	*P*_100;3_ (%)	Median CED_3_ (mSv)	Third quartile CED_3_ (mSv)	Max CED_3_ (mSv)	No. of exams per patient, mean	No. of exams per patient, max	Third quartile CED_1_ in 2022; 2023; 2023 (mSv)
1	48 900	50%	62 [48-75]	129 303	26 651 (55%)	507	0.2	1.0	0.4	5.2	11.4	967.0	2.6	94	10.2; 9.4; 9.6
2	22 980	48%	62 [45-78]	38 363	12 385 (54%)	785	1.0	3.4	2.8	10.7	25.5	1,012.5	2.7	41	15.7; 15.0; 16.0
3	55 209	50%	65 [53-76]	102 204	21 173 (38%)	558	0.2	1.0	0.4	6.8	16.7	639.5	2.0	39	11.5; 11.7; 10.3
4	23 339	48%	69 [57-80]	62 563	12 944 (55%)	1207	0.4	5.2	2.6	12.5	35.9	494.0	2.7	24	19.0; 18.0; 20.0
5	21 847	49%	66 [50-76]	42 537	9442 (43%)	749	0.6	3.4	1.7	10.8	26.5	535.6	2.7	29	15.1; 15.2; 15.0
6	10 430	51%	67 [53-76]	26 735	5798 (56%)	356	0.4	3.4	1.6	12.6	28.6	394.7	2.6	25	16.5; 16.7; 16.6
7	19 132	49%	67 [53-77]	52 742	10 952 (57%)	627	0.4	3.3	1.6	11.2	25.7	548.5	2.8	30	15.9; 15.0; 14.8
8	25 864	53%	64 [51-74]	45 917	9199 (36%)	1676	2.2	6.5	2.1	12.9	36.4	882.9	3.2	27	26.4; 26.6; 25.8
9	36 063	49%	68 [53-80]	86 133	17 968 (50%)	1362	0.7	3.8	1.9	7.5	26.6	555.5	2.4	40	17.3; 17.3; 17.0
10	16 420	45%	67 [56-77]	79 259	8464 (52%)	301	0.3	1.8	0.9	5.6	13.7	513.1	2.5	27	11.2; 12.9; 13.0
11	19 106	43%	61 [47-74]	46 165	8070 (42%)	444	0.7	2.3	0.9	5.0	18.4	531.8	1.9	32	16.5; 13.6; 12.0
12	23 043	47%	65 [49-74]	55 329	12 210 (53%)	1415	0.8	6.1	2.4	16.9	41.6	419.9	2.4	41	25.0; 24.5; 23.5
13	7119	45%	66 [44-78]	11 637	2225 (31%)	336	1.7	4.7	1.7	12.0	33.7	632.9	1.6	17	26.2; 26.3; 25.1
14	33 252	50%	65 [52-74]	50 539	17 084 (51%)	1166	0.5	3.5	1.7	8.3	27.2	584.0	2.4	33	16.4; 15.6; 16.1
15	14 701	51%	65 [54-73]	22 259	4624 (31%)	296	0.6	2.0	1.5	22.1	26.8	361.8	1.4	13	25.9; 25.3; 25.5
16	7864	51%	63 [49-73]	12 996	2195 (28%)	76	0.3	1.0	0.3	17.4	39.0	220.0	1.6	17	17.8; 17.6; 17.1
17	39 760	48%	64 [51-74]	113 738	22 862 (58%)	1279	0.7	3.2	1.8	10.1	27.1	623.5	2.9	47	15.4; 14.9; 15.2
18	9561	51%	72 [59-79]	20 904	4497 (47%)	44	0.1	0.5	0.3	5.6	12.1	309.0	2.2	22	7.6; 8.1; 10.0
19	14 890	50%	70 [56-79]	43 541	8610 (58%)	361	0.3	2.4	1.3	9.8	22.9	338.3	2.9	47	11.7; 13.0; 14.8
20	15 182	51%	70 [53-79]	33 595	7296 (48%)	73	0.1	0.5	0.3	6.2	14.1	279.9	2.2	34	9.3; 9.2; 12.3
21	5372	52%	72 [59-80]	13 890	2936 (55%)	76	0.0	1.4	0.6	7.9	17.5	235.1	2.6	19	8.9; 10.6; 11.8
22	7884	51%	70 [56-78]	17 364	3586 (45%)	81	0.1	1.0	0.5	5.7	13.5	688.2	2.2	27	8.6; 8.6; 9.1
23	23 055	51%	70 [55-79]	64 650	12 980 (56%)	695	0.5	3.0	1.7	10.3	24.2	591.5	2.8	38	14.6; 15.1; 17.6
24	24 287	51%	68 [52-78]	67 895	13 672 (56%)	636	0.3	2.6	1.2	9.2	21.9	627.0	2.8	39	12.8; 12.9; 13.6
25	7901	53%	72 [58-79]	20 827	4233 (54%)	185	0.1	2.3	1.2	9.2	20.3	345.4	2.6	26	12.7; 12.1; 14.7
26	9928	51%	72 [59-79]	29 802	5924 (60%)	233	0.4	2.3	1.1	9.3	21.5	1,189.0	3.0	36	11.5; 11.9; 12.7
27	10 748	53%	66 [51-75]	37 636	2649 (25%)	62	0.3	0.6	0.3	9.8	15.9	437.4	1.3	15	14.9; 15.2; 15.8
28	3767	42%	61 [48-72]	6136	2216 (59%)	261	1.3	6.9	2.3	16.8	38.5	580.7	4.2	27	23.3; 21.4; 19.8
29	10 361	46%	67 [52-79]	22 443	5781 (56%)	0	0.0	0.0	0.0	5.0	10.9	90.6	1.6	21	8.9; 7.5; 7.5
30	24 910	51%	65 [51-74]	41 870	12 091 (49%)	2148	2.6	8.6	4.4	12.9	39.4	670.5	2.2	28	21.8; 27.4; 29.5
31	118 874	51%	67 [52-77]	259 274	65 748 (55%)	2760	0.2	2.1	0.5	6.2	14.6	552.0	2.0	34	12.6; 12.5; 12.3

The last column shows yearly values of third quartile CED (denoted as CED_1_).

A small but statistically significant (*F *= 4.5; *P *= .01) increase in the average number of patients (from 21 960 ± 20 243 in 2022 to 23 353 ± 21 730 in 2024), CT examination per facility (from 33 446 ± 31 966 in 2022 to 36 282 ± 33 788 in 2024, *F *= 6.7; *P *= .002) and *I*_100;1_ (from 0.53% ± 0.58% in 2022 to 0.67% ± 0.65% in 2024; *F *= 3.69; *P *= .03) was found in the years 2022-24 (Figure 1).

**Figure 1:**
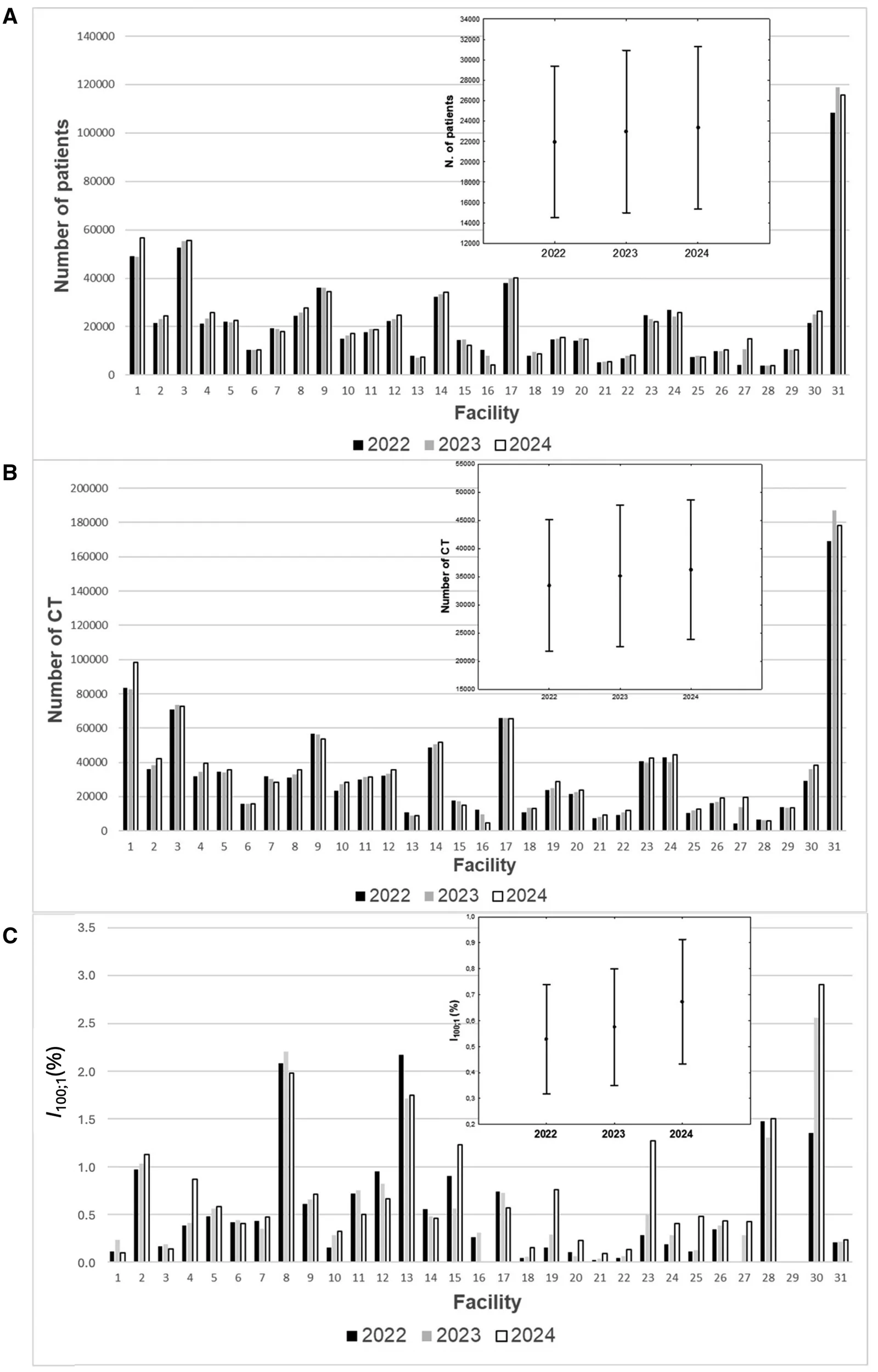
Temporal distribution of (A) number of patients; (B) number of CT; (C) *I*_100;1_ in 2022-24 in different facilities. The top inserts show least square averages and 95% confidence intervals of the mean values among facilities.

Figure 2 presents the box-plot histograms of median *I*_100;1_ for the 3-year period, *I*_100;3_ and *P*_100;3_.The *I*_100;1_ over the 3-year period had a mean value of 0.59% (median 0.43%; IQR = 0.15%-0.75%) and ranged from a minimum of 0% for at least one of the years in facilities #16, 27 and 29, to a maximum of 2.9% in 2024 for facility #30. The *I*_100;3_ had a mean value of 2.9% (median 2.4%; IQR= 1.2%-3.5%) and ranged from a minimum of 0% for facility #29 to a maximum of 8.6% for facility #30. The *P*_100;3_ had a mean value of 1.3% (median 1.3%; IQR= 0.5%-1.7%) and ranged from a minimum of 0% for facility #29 to a maximum of 4.4% for facility #30.

**Figure 2:**
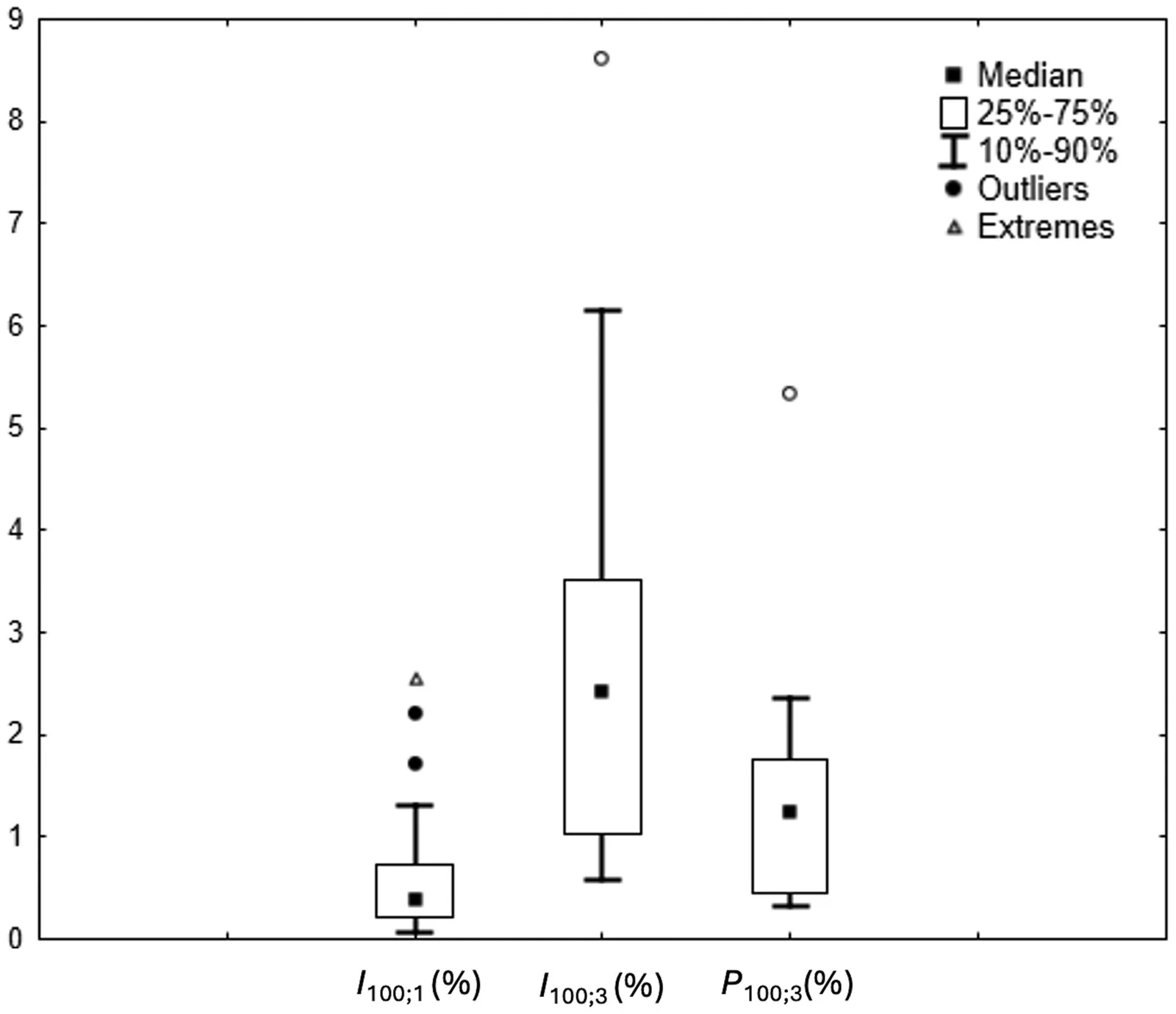
Box plot of histograms of *I*_100;1_, *I*_100;3_, and *P*_100;3_. *I*_100;1_ are the median values registered in 2022-24 in each center.

The percentage of recurrent patients had a mean value of 49% (median 53%; IQR= 44%-56%) ranging from a minimum of 25% for facility #27 to a maximum of 60% for facility #26. No significant correlations were found between the percentage of recurrent patients and *I*_100;3_ (*ρ* = 0.19; *P > .*05) or *P*_100;3_ (*ρ* = 0.21; *P > .*05).

For the entire 3-year period, the correlation between *I*_100;1_ and the third quartile values of yearly CED was significantly higher than the correlation with the median value of yearly CED (*r *= 0.85 vs. *r *= 0.40; *P *= .002).

A strong positive linear relationship was found between the third quartile values of yearly CED and *I*_100;1_ (*R*^2^ = 0.72; *P < *.0001). The linear regression equation is:


$$I_{100;\;1}=0.095*3^{\mathrm{rd}}\mathrm{quartile}\;\mathrm{CED}(\mathrm{mSv})-0.90$$

Figure 3A presents the regression line between *I*_100;1_ and the third quartiles of the yearly CED, and Figure 3B—a plot of the residuals versus the third quartile values of yearly CED, showing absence of any significant correlation (*r *= 0.9 × 10^−8^) between the residuals and the independent variable, which is a condition for validity of the regression model. The normal probability plot of the residuals did not show any significant departure from normality in the distribution of raw residuals. Diagnostic analyses revealed mild heteroscedasticity in the residuals, as indicated by a funnel-shaped residual-versus-predictor plot and a significant Spearman rank correlation between the absolute residual values and predictor values (*ρ* = 0.53; *P < *.01). Nevertheless, heteroscedasticity is not expected to bias the regression coefficients relating *I*_100;1_ to the third quartiles of the yearly CED, which constituted the primary objective of this predictive regression analysis.

**Figure 3:**
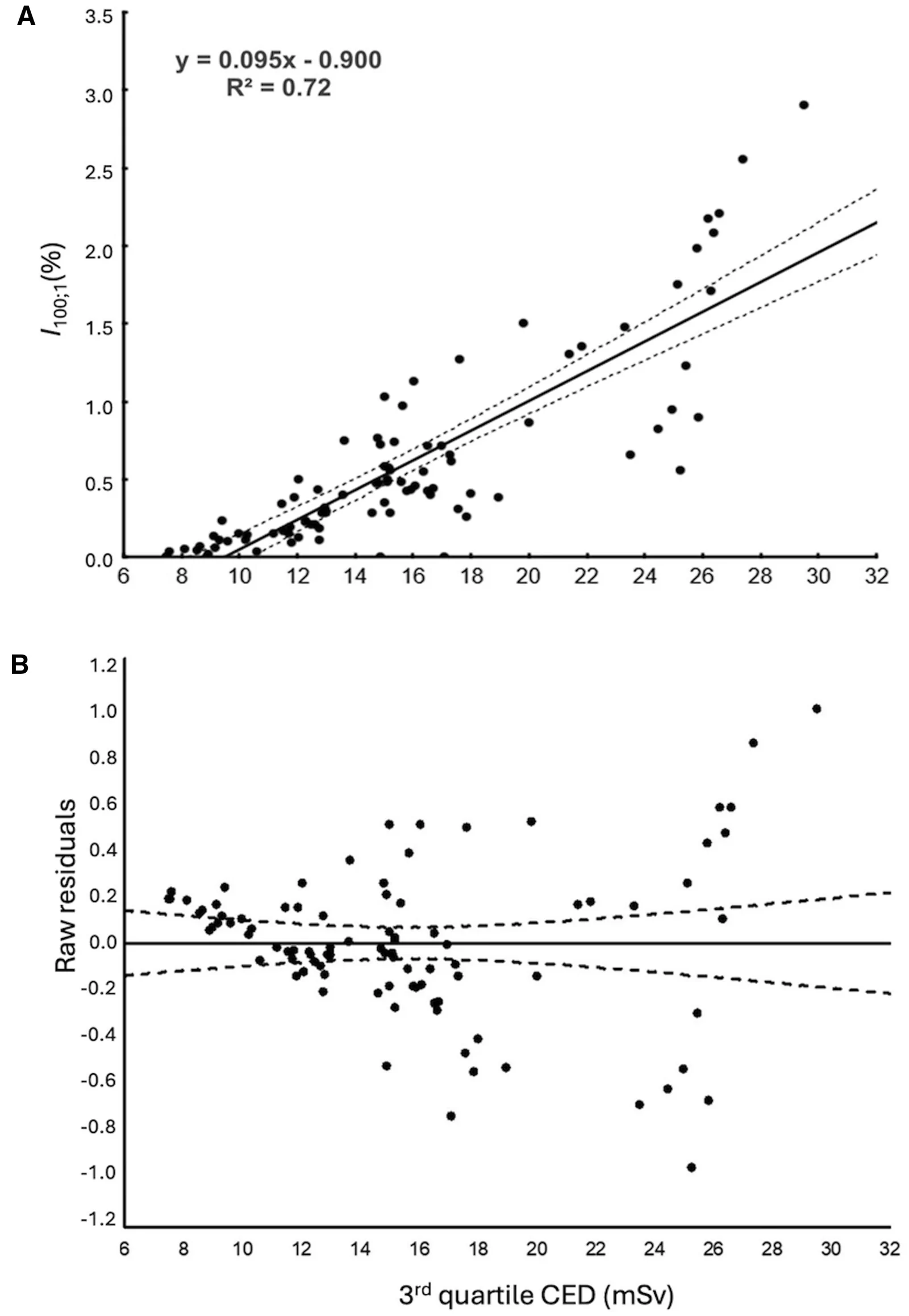
(A) Regression line between *I*_100;1_ and the third quartiles of the yearly CED; (B) plot of the raw residuals versus the third quartile values of yearly CED. Dotted lines are 95% confidence intervals.

A polynomial fitting (Figure 4) provides a higher proportion of variance (76%) accounted for by the quadratic model:


$$I_{100;\;1}=0.0033*{\left[3^{\mathrm{rd}}\mathrm{quartile}\;\mathrm{CED}(\mathrm{mSv})\right]}^{2}-0.020*3^{\mathrm{rd}}\mathrm{quartile}\;\mathrm{CED}(\mathrm{mSv})$$

**Figure 4:**
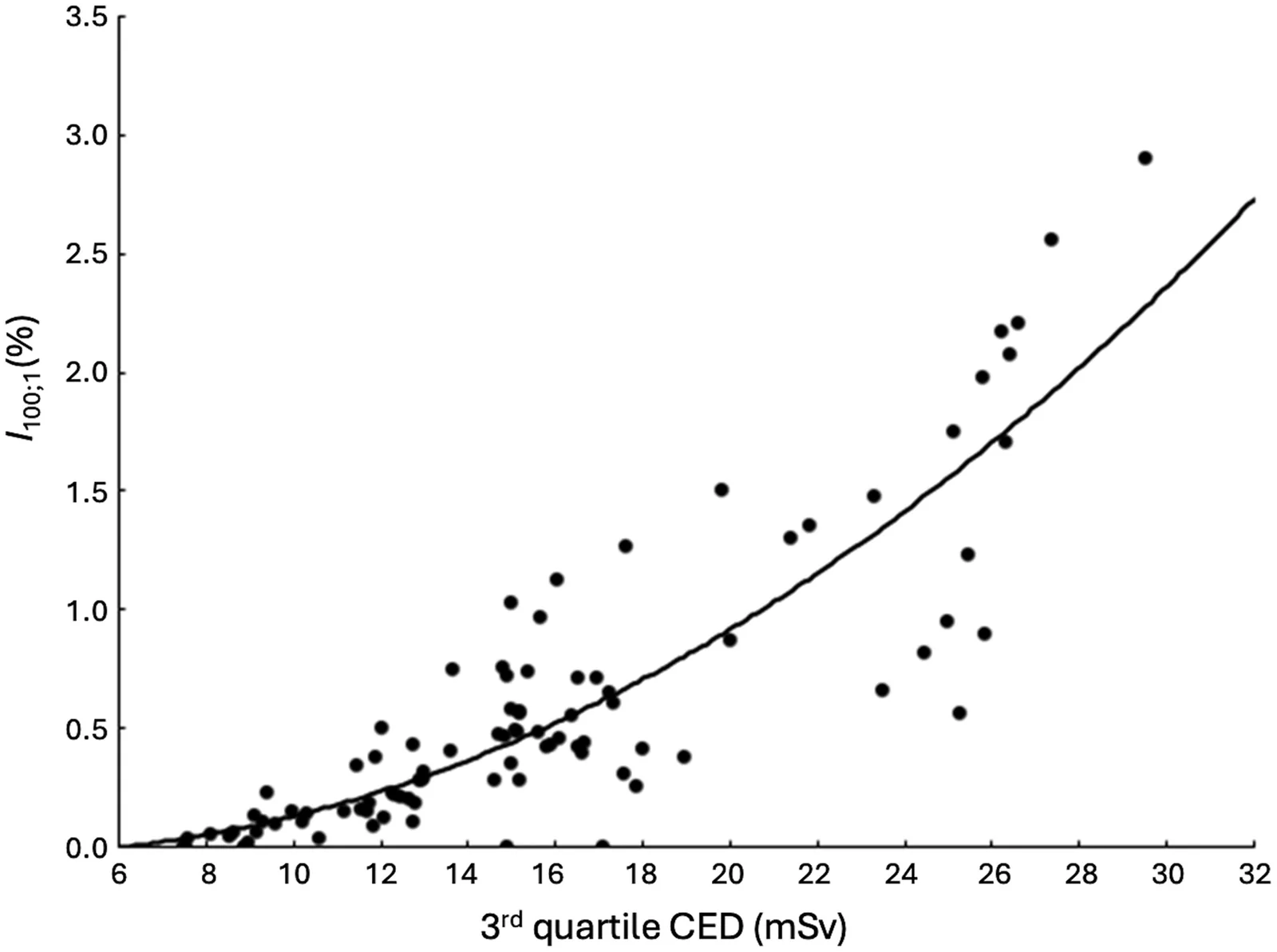
Regression line between *I*_100;1_ and the third quartiles of the yearly CED using a quadratic fitting.

Figure 5 shows histogram of the pooled third quartiles of the yearly CED for years 2022, 2023 and 2023 for this sample of 31 European facilities (93 values shown in the last column of Table 1). The 75th percentile of the distributions amounted to 17.6 mSv, which was exceeded in 7 out of 31 (13%) facilities.

**Figure 5:**
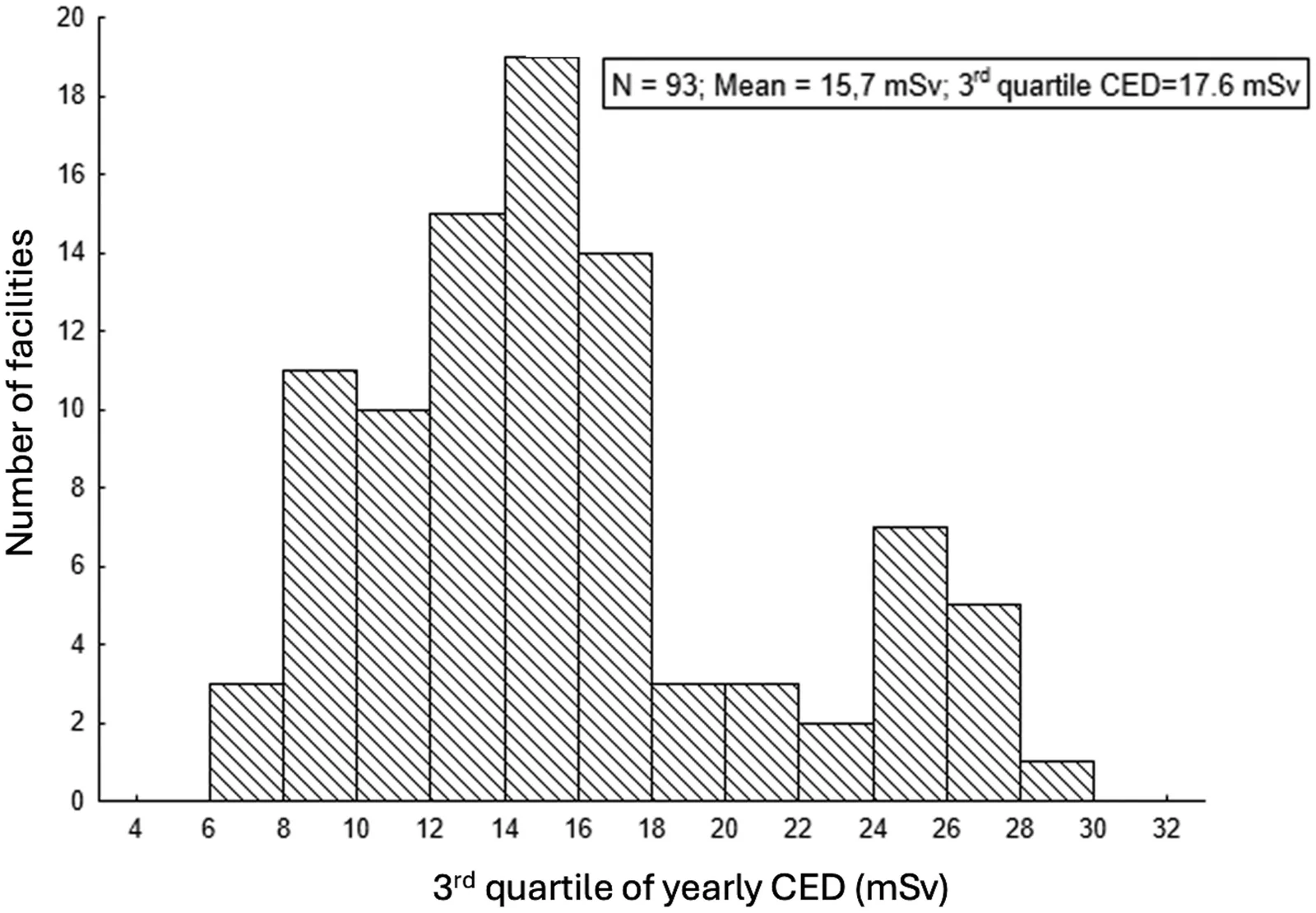
Distribution of facilities’ third quartiles of the yearly CED (mSv).

The regression equation that best summarizes the results obtained in a multiple linear regression model with *I*_100;3_ as the predicted variable and different statistical CED descriptors over 1 year (median CED_1_; third quartile CED_1_, *I*_100;1_, average number of exams per patient), as well as the percentage of recurrent patients as a predictor variable may be written as:


$$I_{100;\;3}=0.33*3^{\mathrm{rd}}\mathrm{quartile}\;\mathrm{CED}(\mathrm{mSv})+7.9*\mathrm{Recurrent}\;(\%)-5.9$$

The multiple $R_{\text{adjusted}}^{2}$ of model fitting was 0.86; third quartile CED (mSv) and the percentage of recurrent patients were retained in the model as statistically significant predictors of *I*_100;3_, which variance can be accounted for, in order of decreasing relevance, by the third quartile CED with a standardized regression coefficient *β*_1_ = 0.95 and a partial $R_{\text{adjusted}}^{2}$ = 0.75, and the percentage of recurrent patients with *β*_2_ = 0.34 and an additional $R_{\text{adjusted}}^{2}$ = 0.11.

The regression equation is represented as a single surface plot in Figure 6.

**Figure 6:**
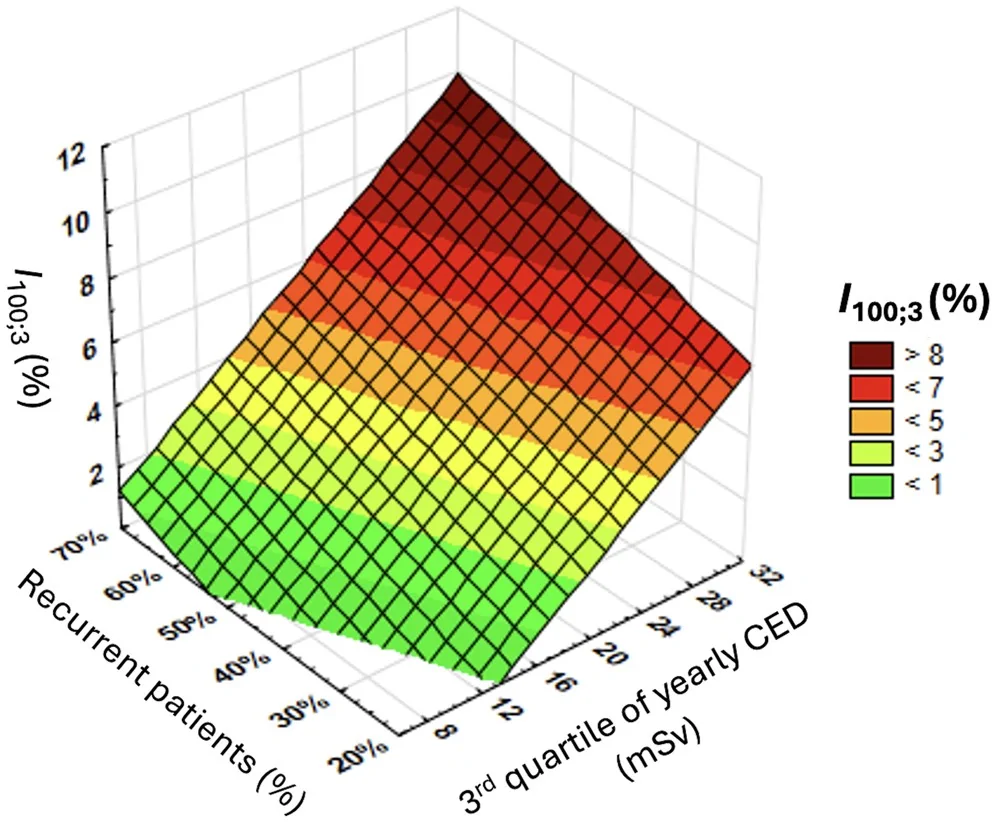
Surface plot with linear fitting of the regression equation relating *I*_100;3_ with third quartile of yearly CED, and percentage of recurrent patients.

## Discussion

This study confirms some hypotheses from the earlier studies^25^^,^^26^ and introduces new findings.

The first confirmation is that there is significant variability among health care facilities of similar type and size in how they manage recurrent patients. *I*_100;1_ in the reference year ranged from 0% to 2.6%, while *I*_100;3_ spanned from 0 in facility #29 to 8.6% in facility #30. This means that in facility #29 no patient is likely to exceed 100 mSv in 3 years, while in facility #30 about 1 in 11 patients will exceed this value. Notably, both facilities are university hospitals with over 500 beds and present similar average numbers of CT scans per patient across 3 years (1.6 versus 2.2). Additionally, the proportion of recurrent patients is higher at facility #29 (56%) than at facility #30 (49%). While an in-depth analysis of the underlying causes lies beyond the scope of this study, these results suggest the need to examine the allocation of indication-specific CT acquisition protocols for recurrent imaging and, if needed, optimize relevant protocols.

The second confirmation concerns the consistent linear relationship between *I*_100;1_ and the third quartile values of yearly CED, which forms the theoretical foundation for using latter metric to set a benchmarking reference level. As shown in Table 2, although different studies report significant variability in the distributions of both *I*_100;1_ and the statistical descriptors of CED, the linear relationship between *I*_100;1_ and the third quartile of yearly CED remains stable, as is the percentage of variance of *I*_100;1_ explained by the linear relationship.

**Table 2:** Comparison of the linear relationship between *I*_100;1_ (%) and third quartile of yearly CED (denoted as CED_1_) in published studies.

Study	No. facilities	Linear relationship between *I*_100:1_ (%) and third quartile of yearly CED	*R* ^2^	*I*_100;1_ (%), mean	*I*_100;1_ (%), range	Third quartile CED_1_, mean (mSv)	Third quartile CED_1_, range (mSv)	RERL (mSv)
Present study	31	*I*_100;1_ = 0.095 *X* − 0.9	0.72	0.58%	[0.0-2.6%]	15.7	[7.5-29.5]	17.6
Brambilla et al (2024),^26^ Slovakia	61	*I*_100;1_ = 0.11 *X* − 1.04	0.71	1.0%	[0.0-8.7%]	21.6	[9.8-60.4]	25.7
Brambilla et al (2024),^25^ Italy	9	*I*_100;1_ = 0.16 *X* − 2.34	0.80	2.0%	[0.1-5.3%]	26.8	[12.7-40.1]	34.0

This study confirms that using prevalence indexes to estimate the proportion of patients who accumulate CED > 100 mSv over several years is methodologically flawed and leads to significant underestimation. Our average *I*_100;3_ value was 2.9%, more than twice the *P*_100;3_ index of 1.3%. Similar trends were observed in 2 other studies: Brambilla et al^25^ reported 6.4% versus 2.6% in Italian hospitals, and Brambilla et al^26^ reported 3.9% versus 2.2% in Slovakian hospitals. Prevalence tends to underestimate the true magnitude because not all patients in its denominator are observed over the same length of time. This is akin to taking a snapshot of an ongoing event rather than capturing its full progression. In contrast, incidence metrics account for observations across varying time periods, making them more like videos that reflect changes over time. The authors recommend using incidence as the preferred metric for future studies assessing recurrent imaging.

While 0.58% of patients with CED > 100 mSv in a single year may be considered negligible in absolute terms, over a 3-year period, the average proportion rises to approximately 3% among all patients undergoing CT scans. With further extension of the observation period, this percentage is projected to increase to 5%-6%.^8^ Future studies should examine this trend for deeper insight into recurrent imaging patterns.

A new finding of this study is the multiple linear relationship that associates *I*_100;3_ with statistically significant predictors. The link between the third quartile of yearly CED and *I*_100;1_ was anticipated, since the shape of each center’s CED distribution determines its relationship with *I*_100;1_. Furthermore, the third quartile of yearly CED is the most important predictor of *I*_100;3_. This means that using a single dose index-the third quartile of yearly CED—can provide reasonably reliable estimates of the temporal trend of the percentage of patients likely to exceed 100 mSv over a 3-year period. The only other statistically significant predictor of *I*_100;3_ is the percentage of recurrent patients. Combined, these 2 predictors account for 86% of the observed variation among facilities regarding the 3-year incidence of patients with CED > 100 mSv.

While some may argue that using retrospective recurrence rates makes predicting *I*_100;3_ from single-year data unreliable, these rates typically remain stable in the same facility over several years.

It is noteworthy that the annual number of CT scans per patient does not predict either *I*_100;1_ or *I*_100;3_. This indirectly suggests that the high values of *I*_100;1_ and *I*_100;3_ are primarily associated with the dose from a single CT scan, which may encompass varying numbers of acquisition sequences, and the percentage of patients undergoing repeated examinations, rather than with a particularly high mean number of CT examinations per patient.

The study has some limitations. Voluntary participation was limited to European centers with strong quality assurance and qualified medical physicists, leading to positive selection bias and potentially underestimating results. Patient tracking was restricted to single facilities, so cumulative dose data may be incomplete for those scanned at multiple sites. Only CT exams were included, possibly resulting in an underestimated total CED as many recurrent patients might also undergo interventional radiology, nuclear medicine, and other imaging studies.^10^ Future research could apply the methodology of this study to investigate recurrent imaging in multi-modality settings.

A single DLP-to-ED conversion per anatomical region did not account for patient size, age, sex, which is considered acceptable for population-level benchmarking and optimization studies when individual risk assessment is outside the objectives. Standardized DLP-to-ED conversion coefficients were used to minimize inconsistencies in organ dose and ED calculations, thereby improving reproducibility. Expected improved methods for assessment of ED and their standardized utilization across REMSs may contribute to improved CED assessment in future.

This study was not designed to investigate factors contributing to varying recurrent rates; its main goal was to propose a strategy for objectively evaluating the local situation as an initial step in the optimization process. By following the methodology outlined here, a medical facility can track their recurrent patients over at least 3 years, calculate the third quartile of yearly CED values, and then compare this figure with similar values from other facilities, or with the reference level if established. A higher value does not immediately signal suboptimal recurrent imaging practice, as it could be explained by a specific clinical context or complexity of diseases managed by the facility. However, such higher values could result from excessive use of CT to follow-up of patients with chronic conditions, inadequate justification for imaging procedures, utilization of suboptimal CT protocols, and other factors. Though this may not have an immediate impact on individual patient management, investigating the contributing factors and addressing high recurrent rates can ultimately improve patient care.
